# Analysis of Cannabinoids and Metabolites in Dried Urine Spots (DUS)

**DOI:** 10.3390/molecules26175334

**Published:** 2021-09-02

**Authors:** Matteo Moretti, Francesca Freni, Claudia Carelli, Carlo Previderé, Pierangela Grignani, Claudia Vignali, Maria Cobo-Golpe, Luca Morini

**Affiliations:** 1 Department of Public Health, Experimental and Forensic Medicine, University of Pavia, Via Forlanini 12, 27100 Pavia, Italy; matteo.moretti19@gmail.com (M.M.); frafre93@gmail.com (F.F.); claudia.carelli01@universitadipavia.it (C.C.); carlo.previdere@unipv.it (C.P.); pierangela.grignani@unipv.it (P.G.); claudia.vignali@unipv.it (C.V.); 2 Servizo de Toxicología, Instituto de Ciencias Forenses, Universidade de Santiago de Compostela, San Francisco s/n, 27002 Santiago de Compostela, Spain; m.cobo@usc.es

**Keywords:** dried urine spots, cannabinoids, THC, THCCOOH, THCCOOH-gluc, LC-MS/MS

## Abstract

Dried urine spots (DUS) represent a potential alternative sample storage for forensic toxicological analysis. The aim of the current study was to develop and validate a liquid chromatographic tandem mass spectrometric procedure for the detection and quantitative determination of cannabinoids and metabolites in DUS. A two-step extraction was performed on DUS and urine samples. An LC-MS/MS system was operated in multiple reaction monitoring and positive polarization mode. The method was checked for sensitivity, specificity, linearity, accuracy, precision, recovery, matrix effects and carryover. The method was applied to 70 urine samples collected from healthy volunteers and drug addicts undergoing withdrawal treatment. The method was successfully developed for DUS. LODs lower than 2.0 ng/mL were obtained for all the monitored substances. All the validation parameters fulfilled the acceptance criteria either for DUS or urine. Among the real samples, 45 cases provided positive results for at least one compound. A good quali-quantitative agreement was obtained between DUS and urine. A good stability of THC, THCCOOH and THCCOOH-gluc was observed after a 24 h storage, in contrast to previously published results. DUS seems to provide a good alternative storage condition for urine that should be checked for the presence of cannabinoids and metabolites.

## 1. Introduction

Dried matrix spots (DMS) are increasingly being used for numerous applications in the field of clinical and forensic toxicology.

This technique consists of taking and depositing a small amount of a biological fluid (blood, urine, sweat, etc.) onto a filter card and letting it dry. The main advantages and disadvantages of DMS are summarized in Table 1.

Since the 1950’s, several methods have been developed and validated for dried blood spots [1,2,3,4,5,6]. Among DMS, dried urine spots (DUS) have successfully been used for several analytical purposes. In 1959, DUS was used to detect specific proteins in urine, in order to diagnose diseases such as phenylketonuria [7]. The use of DUS was then adapted in the following years to search for other proteins and metabolites [8,9,10,11,12].

Since urine is one of the most frequently analyzed matrices in forensic toxicology, DUS have recently been investigated as potential alternative samples for the identification and quantification of drugs and metabolites. Some methods have been developed for different drug classes [13,14,15]. In particular, Lee and coauthors [14] developed a method for the detection and quantification of 19 drugs of abuse and metabolites. Their method focused on the identification of substances that can easily be ionized in positive polarization, such as opiates, opioids, cocaine and amphetamine derivatives. However, the authors proved that also 11-nor-9-carboxy-Δ9-THC (THCCOOH), the main metabolite of THC that is generally ionized in negative mode, can be detected on spiked DUS. Pablo et al. [15] developed an automated LC-MS/MS screening procedure that was tested for 41 compounds among drugs of abuse, medicines and metabolites. The authors observed a scarce stability of THCCOOH on DUS, thus leading to unreliable quantitative results a few hours after deposition time. This fact represents a major weakness for the potential use of DUS in forensic toxicology, especially in cases involving the detection of cannabinoids.

To date, however, no specific and fully validated methods for the identification and quantitative determination of different cannabinoids and their metabolites in urine have been published.

The aim of this study was the development and validation of an LC-MS/MS method for the simultaneous evaluation of three phytocannabinoids and three main metabolites of THC in DUS and urine, and the assessment of the agreement between results in the two types of samples.

## 2. Results

The analytical procedure was fully validated for THC, CBG, CBD and three metabolites of THC, namely, 11-OH-THC, THCCOOH and THCCOOH-gluc. The identification of CBN was evaluated only for qualitative purposes, due to the lack of a certified standard. Chromatograms of a blank sample, a spiked sample at LOQ level and of a real positive sample are reported in the Figures S1–S3 Supplementary Material.

Methanol was chosen as the extraction solvent for several reasons. It is a relatively low-cost solvent; it is less toxic than others (such as chlorinated organic solvents); and the laboratory is very experienced in extraction procedures, including the extraction of phytocannabinoids from biological matrices based on methanol. 

Recovery was evaluated at different sonication times (5, 10, 15, 20, 25 and 30 min). Fifteen minutes was considered as the best choice in terms of time saving and recovery.

Different solvents were tested for liquid/liquid extraction (LLE) optimization. Initially, hexane and a mixture of hexane-ethyl acetate (9:1) were tested, since cannabinoids are generally extracted by means of these two solvents. However, though matrix effects were relatively negligible, a recovery lower than 40% was achieved. Then, a mixture of diethyl ether-ethyl acetate (1:1, *v*/*v*) was tested. Since a recovery close to 100% was observed, no further experiments were performed, and the latter mixture was chosen for method development. Despite the small amount of sample (25 µL), the method was assessed to be sensitive and specific for all the analytes. All the LODs and the LOQs are listed in Table 2.

The method was found to be linear in the range 10–400 ng/mL. The coefficients of determination (r^2^) calculated for the curves were higher than 0.99. Accuracy (expressed as Bias%) and imprecision (expressed as CV%) calculated at three quality control levels were always lower than 14.3% and 16.0%, respectively. All data are reported in Table 3.

The two consecutive extraction procedures increased the extraction efficiency of all six analytes from the filter cards. A recovery always higher than 85.0% was measured for all monitored substances. Matrix effects were found to be negligible at all three quality control levels. Carryover phenomena were not observed in blank chromatograms carried out after C6 calibrator (400.0 ng/mL), and, only for THCCOOH-gluc, after the injection of a spiked sample at the concentration of 2000.0 ng/mL.

Among the 70 analyzed urine samples, all 45 positive samples determined by the enzyme multiplied immunoassay technique (EMIT^®^, Siemens Healthcare, Milan, Italy) were then confirmed both in urine and DUS. Moreover, none of the analytes were observed in the remaining 25 samples.

Only THC, THCCOOH and THCCOOH-gluc were detected and quantified in the positive samples. In particular, THCCOOH and THCCOOH-gluc were found in all 45 samples, while the presence of THC was identified in 30 out of 45 urine samples. THC concentrations on DUS ranged from 10.5 to 61.5 ng/mL (median: 14.4 ng/mL; mean: 17.3 ng/mL; *n* = 17), while THCCOOH was quantitated in the range 10.0–232.0 ng/mL (median 18.2 ng/mL; mean: 35.2 ng/mL; *n* = 45), and THCCOOH-gluc in the range 19.7–1840.0 ng/mL (median: 84.2 ng/mL; mean: 226.2 ng/mL; *n* = 45).

A good qualitative and quantitative correlation was observed between results obtained in urine and in DUS. The urine and DUS concentrations significantly correlated for THC (Spearman’s rs 0.74584), THCCOOH (Spearman’s rs 0.89516) and THCCOOH-gluc (Spearman’s rs 0.93939).

**Figure 1:**
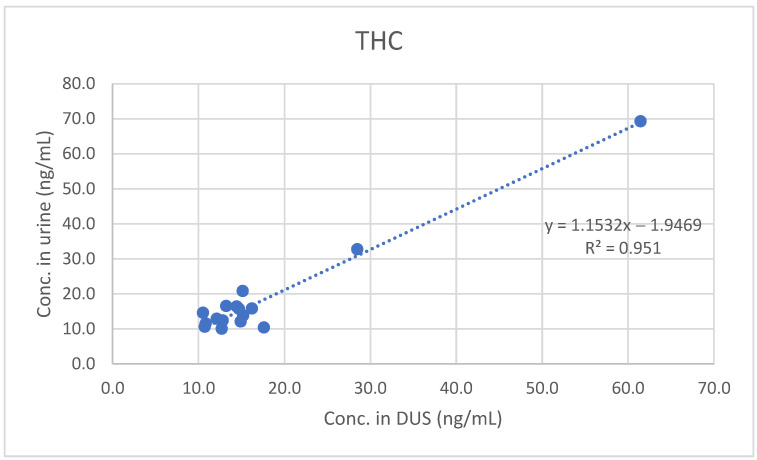
Comparison between urine and DUS concentrations of THC.

**Figure 2:**
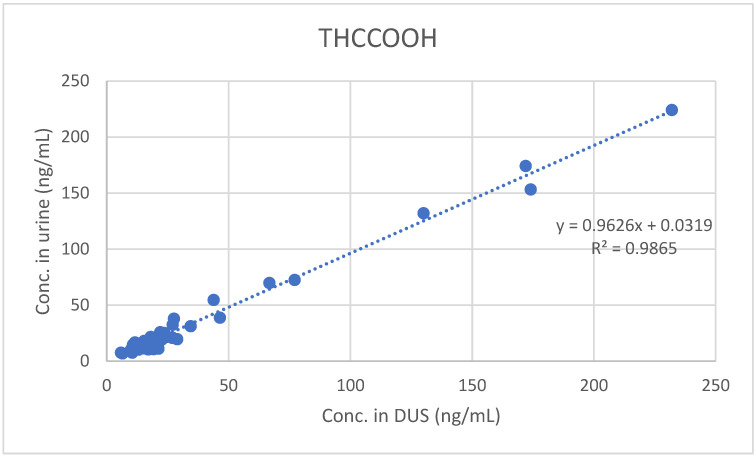
Comparison between urine and DUS concentrations of THCCOOH.

**Figure 3:**
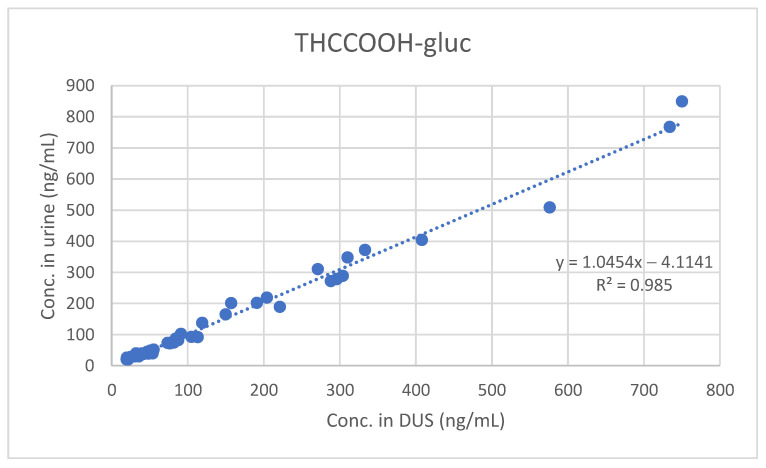
Comparison between urine and DUS concentrations of THCCOOH-gluc.

Furthermore, least-squares regression analysis confirmed the significant quantitative correlation between concentrations measured in urine and those measured in DUS (see Figure 1, Figure 2 and Figure 3).

**Figure 4:**
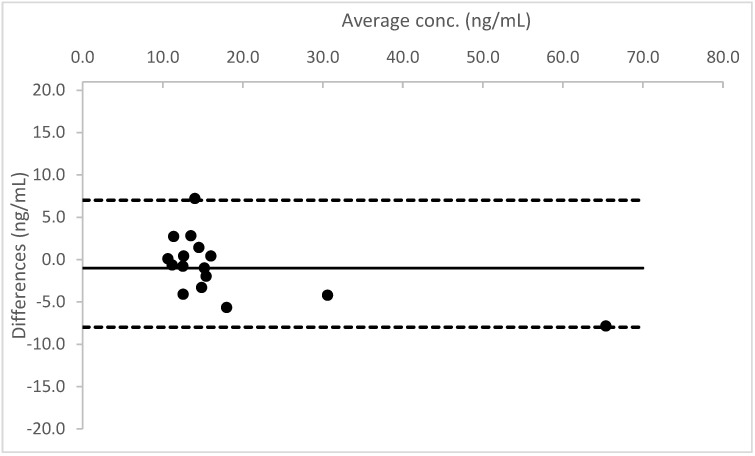
Bland–Altman plot for the results obtained from Whatman™ 903 card, in comparison to the data measured in liquid urine for THC.

**Figure 5:**
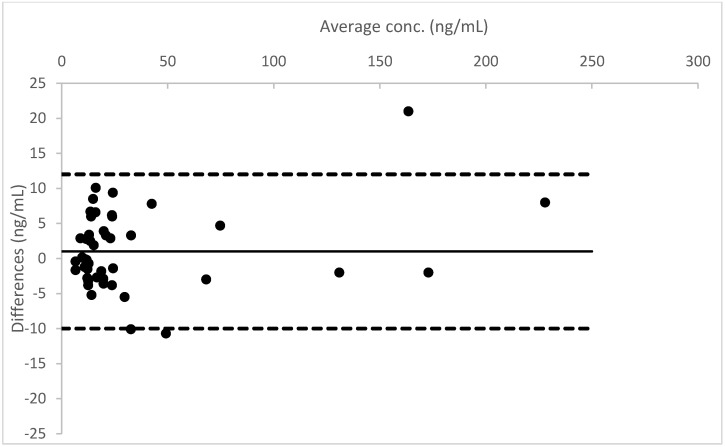
Bland–Altman plot for the results obtained from Whatman™ 903 card, in comparison to the data measured in liquid urine for THCCOOH.

**Figure 6:**
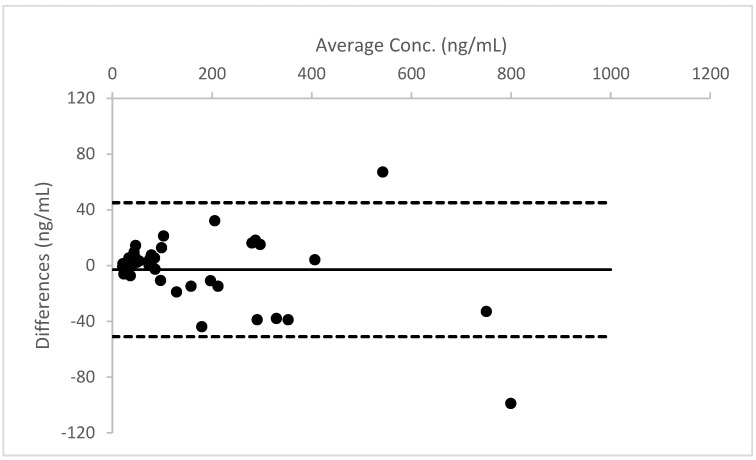
Bland–Altman plot for the results obtained from Whatman™ 903 card, in comparison to the data measured in liquid urine for THCCOOH-gluc.

A Bland–Altman plot, performed for all three analytes, proved good agreement between the two different urine measurements (see Figure 4, Figure 5 and Figure 6).

## 3. Discussion

The analytical method was successfully developed and validated on urine and DUS. Initially, a single extraction procedure was evaluated; methanol could extract THC, CBD, CBG and 11-OH-THC at a relatively high extraction efficiency rate. However, recovery rates for THCCOOH and THCCOOH gluc proved inadequate. Hence, a further extraction procedure, using a buffer solution at pH 4.5 followed by a liquid/liquid extraction with diethyl ether-ethyl acetate mixture (1:1, *v*/*v*), allowed an increase in THC carboxy metabolites recovery. Though diethyl ether is normally avoided to prevent physical hazards, it provided the best results in terms of extraction efficiency and matrix effects. A good sensitivity was achieved for all six analytes, as reported in Table 2. Since the main objective of the study was to evaluate the agreement between results in urine and in DUS, it was decided to set the calibration curve within the range 10–400 ng/mL. However, the experiments carried out during LOQ evaluation confirmed that, if required, quantification could be performed up to the 1 ng/mL level for THC and up to the 5 ng/mL level for the other compounds.

In total, 45 out of 70 real cases provided positive results for at least one analyte. The LC-MS/MS analyses performed on DUS and urine confirmed the qualitative results obtained by the immunoassay screening method. THCCOOH and THCCOOH-gluc were identified and quantified in all 45 positive samples. Among the 17 THC positive samples, only two cases provided a concentration higher than 20 ng/mL. Though the absence of 11-OH-THC in all of the positive samples could be due to its relatively low sensitivity, previously published studies, achieving a higher sensitivity [16,17], did not detect this metabolite in urine either. Additionally, CBD and other phytocannabinoids were not observed in real samples. The main reason could be due to the consumption by the monitored subjects of cannabis-based products with high levels of THC rather than legal cannabis products, which usually contain high levels of CBD and/or other cannabinoids. In order to prove the reliability of the method, the LC-MS/MS procedure was checked on urine samples collected by four users of legal cannabis-based products (cannabis with THC concentrations lower than 0.5% and percentages of CBD higher than 5%). CBD was identified and quantified in all urine samples. The results were not included in the study because the samples were part of another research project, and urine samples of the four subjects were not deposited on DUS.

To the best of our knowledge, our study is the first to focus on the evaluation of cannabinoids and metabolites in DUS. The comparison between DUS and urine samples found a good qualitative and quantitative correlation. In particular, all the analytes found in urine were also detected in DUS. Moreover, the concentrations of THC, THCCOOH and THCCOOH-gluc measured in urine did not significantly differ from those measured in urine. The measurement of THCCOOH-gluc is of interest because it could be useful to evaluate recent cannabis-based product consumption. In fact, as suggested by previously published studies, the measurement of phase II metabolites may provide important information about the estimated elapsed time from the last consumption among chronic cannabis users. Unfortunately, we could not obtain a reference standard of THC-gluc, another important phase II metabolite of THC.

Moreover, in contrast to previously published results [15], this study proved that THC, THCCOOH and THCCOOH-gluc are stable on DUS for at least 24 h. The reason for this discrepancy between results obtained from the present work and the published one could be due to the different extraction procedure adopted. Indeed, Pablo et al. stated that the loss of THCCOOH signal in spiked samples after a few hours following deposition could be caused either by a degradation of the compound on DUS or by a limited extraction efficiency of the developed sample treatment. The two-step extraction that was adopted in this study supported the hypothesis that cannabinoids are stable, at least for 24 h, when stored on a dried biological matrix in the dark at room temperature, but also that sample preparation should achieve an excellent extraction efficiency to prevent signal loss.

The main drawback of the study is the lack of data concerning the stability of cannabinoids in DUS. Previous studies have observed that THC and metabolites in urine can undergo degradation when they are stored at room temperature [18,19,20]. It will be important to monitor stability to assess the reliability of DUS over a long time. The amount of sample to be deposited on filter papers is relatively low; we decided to use 25 µL of urine. As observed during validation, this issue has a negative impact on method sensitivity, and could lead to false negative results.

## 4. Materials and Methods

### 4.1. Chemicals

Delta-9-tetrahydrocannabinol (THC), 11-nor-delta-8-tetrahydrocannabinol-9-carboxylic acid (THC-COOH), 11-hydroxy-delta-9-tetrahydrocannabinol (11-OH-THC), 11-nor-delta-9-tetrahydrocannabinol-9-carboxylic-acid-glucuronide (THC-COOH-gluc), cannabidiol (CBD), cannabigerol (CBG) and delta-9-tetrahydrocannabinol-D3 (THC-D3) were purchased from Cerilliant (Milan, Italy); 11-nor-delta-9tetrahydrocannabinol-9-carboxylic-acid-D3 (THC-COOH-D3) was purchased from Lipomed AG (Arlesheim, Switzerland); LC-MS grade formic acid, methanol, acetonitrile, ethyl acetate, diethyl ether and isopropanol were obtained from Carlo Erba SRL (Milan, Italy); water was purified by filtering deionized water on a Milli-Q filtration system from Merck Millipore (Milan, Italy). Cards for DUS (four-spot cards, Whatman 903TM) were purchased from Sigma-Aldrich (Milan, Italy).

### 4.2. Instrumentation

The analyses were performed with an Agilent 1290 system (Agilent Technologies, Palo Alto, CA, USA). The injector needle was externally washed with methanol (3 s) prior to any injection. A Kinetex C18 column (100 × 2.1 mm i.d., 2.6 µm particle size) (Phenomenex, Castelmaggiore, BO, Italy) was kept at 35 °C during the analysis. The mobile phase consisted of bidistilled water with 0.1% formic acid (A) and acetonitrile with 0.1% formic acid (B). A gradient elution, with a constant flow of 0.25 mL/min, was set as follows: 80% A maintained for 1 min, from 80% to 40% within 1 min and from 40% to 15% within 1 min, 15% A maintained for 5 min, re-equilibrated to 80% A for 4 min. Mass spectrometric detection was performed on a 4000 Q-TRAP (AB SCIEX, Foster City, CA, USA). The ESI source settings were as follows: ion-spray voltage, +5000 V; source temperature, 500 °C; curtain gas, 15 psi; nebulization and heating gas (air), 30 psi and 35 psi, respectively. Multiple reaction monitoring (MRM) was optimized using nitrogen as a collision gas (with pressure set at level 8) and a dwell time of 60 ms. Two transitions for each substance were chosen for identification; the most intense was used for quantification purposes. All the transitions and the optimized parameters are listed in Table 4. Data acquisition and elaboration were performed by Analyst software (version 1.6, AB SCIEX, Foster City, CA, USA).

### 4.3. Sample Preparation

Aliquots of 25 µL of urine were pipetted onto the filter cards and left to dry (for about two hours), in the dark, at room temperature. Urine spots were analyzed within 24 h of the deposition, by keeping them in the dark, at room temperature.

Twenty-four hour stability and long-term stability were not evaluated since the main purpose of the study was to assess sample stability at room temperature.

For each spot, the whole stain (a disk with a diameter of about 13 mm) was cut into small pieces and put into a glass tube, containing 1 mL of methanol with deuterated internal standards (THC-D3 and THCCOOH-D3) at a concentration of 2 ng/mL. The solutions were sonicated for 15 min, vortexed for 10 s and centrifuged at 4000× *g* for 5 min. Then, the methanol was separated into a different glass tube and evaporated under a nitrogen stream. An amount of 1 mL acetate buffer solution (pH 4.5) was added to the solid remaining on the filter paper. The sample was sonicated for 15 min, vortexed for 30 s and centrifuged at 5000 RPM for 4 min, following the same procedure described above. A liquid/liquid extraction was performed with the addition of 3 mL of diethyl ether-ethyl acetate mixture (1:1, *v*/*v*). Supernatant organic solutions were separated from the filter cards and added to the same glass tube where the methanol solution was evaporated. Organic solvents were dried under a nitrogen stream, and finally reconstituted in 30 µL isopropanol and 20 µL methanol.

An amount of 25 µL of the same urine samples was contextually treated using the same sample preparation procedure, in order to correlate results obtained from DUS with those measured in urine.

An amount of 5 µL was finally injected in the LC-MS/MS system.

### 4.4. Validation

Limits of detection (LOD) were measured by evaluating the signal/noise (S/N) ratio of three replicates of spiked blank samples at a concentration of 5 ng/mL. A peak with an S/N ≈ 3 was calculated from the results obtained with samples spiked at 5 ng/mL. Eventually, an adequate chromatography and acceptable ion ratio was confirmed on samples spiked at the calculated LOD level. LOQs were fixed at administratively defined decision points, except for those providing less sensitivity. The LOQs were calculated on ten fortified blank samples collected from different sources; all the blank samples were injected in triplicate and the detection, identification, bias, and precision criteria were evaluated.

Urine samples were spiked with working solutions before deposition on filter cards. Working solutions were freshly prepared in methanol at 6 different concentrations. Since the CBN standard was not certified for quantitative determination, this cannabinoid was evaluated only for identification. On the contrary, the analytical procedure was fully validated for the other six analytes. Furthermore, 15 µL of working solutions was added to calibration points in order to achieve a final concentration in urine for all analytes: C1: 10 ng/mL; C2: 20 ng/mL; C3: 50 ng/mL; C4: 100 ng/mL; C5: 200 ng/mL; C6: 400 ng/mL. A 1:10 dilution factor was evaluated and accepted for quantitative determination. Concentrations exceeding 4000 ng/mL were reported as above the upper limit and were not considered in the statistics.

Quality control (QC) samples were prepared by a different operator by independent dilution at concentrations of 20, 75 and 250 ng/mL, respectively. All standard solutions were stored at −20 °C until analysis. A total of 50 blank samples, collected from laboratory staff, after informed consent, were used for the method development, validation and sample measurements. Twenty blank urine samples were deposited on the paper substrate and analyzed for possible interfering peaks during the first step of method validation. A methanolic solution containing more than 100 drugs among commonly prescribed drugs, drugs of abuse and metabolites, at the final concentration of 1000 ng/mL, was added to the urine samples. Selectivity was evaluated at two levels (10 and 400 ng/mL). Linearity was verified by processing 10 calibration curves analyzed on 5 different days, over the whole range. Acceptance criteria included a coefficient of determination (r^2^) > 0.99, and residuals within 3 standard deviations were considered adequate.

Intraday imprecision, expressed as the coefficient of variation (CV%), was calculated analyzing the QC samples in five replicates, while interday imprecision was measured analyzing the QC samples on five different days. The concentration of the analytes in the QC samples was calculated versus the daily calibration curves. Accuracy was determined as the error between the measured value at QC levels and the target concentration. Blank DUS, urine samples (collected from five different sources) and water solutions were spiked at three levels (20, 75 and 250 ng/mL) before and after sample treatment; the absolute peak areas were compared in order to evaluate recovery and matrix effects, respectively. Experiments were carried out in quintuplicate. Recovery and matrix effects were expressed as the percentage of the mean deviation of drug response in DUS and urine samples against the response measured in the mobile phase at the same concentration level. Matrix effects were considered negligible when the peak area ratios were within 20% variability. Recovery was expressed as the extraction efficiency percentage. Carryover was evaluated by means of injection of a blank sample after a urine sample fortified at the concentration of 400 ng/mL and processed following the procedure described above. Only for THCCOOH-gluc, since the highest concentration measured on DUS was 1840.0 ng/mL, we evaluated the potential carryover up to 2000.0 ng/mL.

### 4.5. Application on Real Samples

A total of 70 urine samples were collected (after informed consent acquisition) from drug addicts under withdrawal treatment at local addiction centers. All the samples had been previously anonymized and screened through EMIT^®^ routinely used in the laboratory (lowest calibration point: 20 ng/mL; cut-off: 50.0 ng/mL). Among the 70 chosen samples, 45 provided positive results for cannabinoid, while 25 were negative.

Drug-free urine samples were used for method validation and were obtained from laboratory staff volunteers. For all cases, urine samples were collected in a clean sealed polyethylene vial.

## 5. Conclusions

We successfully developed and validated an LC-MS/MS procedure for the detection and quantitative determination of phytocannabinoids and three different THC metabolites on DUS and urine. A good agreement between results obtained on urine and DUS suggested that paper cards could be a good alternative for urine storage and forensic toxicology analysis. An evaluation of 24 h stability, processed sample stability and long-term stability, especially freeze-thaw stability, will be conducted in future studies.

The study should now be applied to a larger cohort of samples, in order to confirm the good quantitative reliability of the toxicological results. Moreover, the stability of cannabinoids and their metabolites should be investigated within a longer window of time.

## Figures and Tables

**Table 1:** Advantages and disadvantages of DMS.

Advantages	Disadvantages
small sample volume requirements; easy sampling; improved stability of several analytes; low transport and storage costs; reduced infection risks.	the small volume sample requires a sensitive and specific assay for detection and quantification of analytes; possible inhomogeneous distribution of the sample on the paper.

**Table 2:** Method sensitivity.

Analyte	LOD (ng/mL)	LOQ (ng/mL)
THC-CBD	0.3	10.0
CBG	1.1	10.0
CBN	1.4	10.0
THCCOOH	1.4	10.0
THCCOOH-gluc	0.8	10.0
11-OH-THC	1.9	10.0

**Table 3:** Validation parameters of all the detected substances.

	Accuracy		Precision		Matrix Effect	Recovery
	Intraday (Bias%)	Interday (Bias%)	Intraday (CV%)	Interday (CV%)	(%)	(%)
THC						
20 ng/mL	8.3	3.6	4.1	6.2	84.6	86.5
75 ng/mL	0.3	8.9	10.4	8.8	113.3	85.0
250 ng/mL	5.6	3.2	7.4	6.6	80.8	86.0
CBD						
20 ng/mL	0.4	12.1	12.9	9.5	89.9	85.4
75 ng/mL	4.4	6.2	14.5	7.4	85.1	112.8
250 ng/mL	3.8	3.5	6.2	5.4	104.5	113.4
CBG						
20 ng/mL	9.9	2.4	5.9	6.9	91.8	109.70
75 ng/mL	1.5	11.2	13.3	10.3	88.8	90.27
250 ng/mL	11.5	3.0	4.5	5.5	90.3	90.7
THCCOOH						
20 ng/mL	10.7	8.1	3.9	11.5	100.5	92.0
75 ng/mL	0.5	4.7	10.2	16.0	92.8	89.6
250 ng/mL	6.3	5.7	13.8	10.4	92.6	86.9
11-OH-THC						
20 ng/mL	4.0	6.9	8.2	11.7	99.9	96.7
75 ng/mL	0.4	2.3	7.3	14.3	91.7	89.8
250 ng/mL	9.1	4.0	3.1	1.2	108.9	91.1
THCCOOH-gluc						
20 ng/mL	11.1	1.2	12.4	9.5	91.5	88.9
75 ng/mL	8.7	14.3	9.5	13.2	88.3	110.9
250 ng/mL	2.0	7.5	10.1	5.2	98.6	111.1

**Table 4:** MRM parameters. Quantifying transitions are marked in bold.

Analyte	Q1 (*m*/*z*)	Q3 (*m*/*z*)	DP (V)	EP (eV)	CE (eV)	CXP (eV)
**THC-CBD**	**315.4**	**193.3**	**90**	**12**	**30**	**4**
THC-CBD	315.4	259.3	90	12	27	6
THC-CBD	315.4	93.4	90	12	37	15
**CBG**	**317.2**	**193.1**	**90**	**10**	**30**	**10**
CBG	317.2	123.0	90	10	30	15
**CBN**	**311.2**	**222.8**	**90**	**10**	**30**	**15**
CBN	311.2	241.0	90	10	30	15
**THCCOOH**	**345.4**	**299.4**	**130**	**10**	**28**	**11**
THCCOOH	345.4	327.5	130	10	23	11
**THCCOOH-gluc**	**521.5**	**345.0**	**101**	**10**	**21**	**11**
THCCOOH-gluc	521.5	327.0	101	10	25	15
**11-OH-THC**	**331.6**	**193.3**	**96**	**10**	**39**	**11**
11-OH-THC	331.6	133.2	96	10	35	11
**THC-D3**	**318.4**	**196.2**	**90**	**12**	**33**	**6**
THC-D3	318.4	123.0	90	12	30	6
**THCCOOH-D3**	**348.1**	**302.1**	**135**	**10**	**30**	**15**
THCCOOH-D3	348.1	196.1	135	10	30	15

Q1: quadrupole 1; Q3: quadrupole 3; DP: declustering potential; EP: entrance potential; CE: collision energy; CXP: cell exit potential. The transition in bold type was used as a quantifier, and the others as qualifiers.

## Data Availability

Not applicable.

## Supplementary Materials

The following are available online, Figure S1: Individual MRM transitions of blank DUS sample. (A) MRM transition 318.4/196.2 of THC-D3; (B) MRM transition 315.4/193.3 of THC and CBD; (C) MRM transition 345.4/299.4 of THCCOOH; (D) MRM transition 521.5/345.0 of THCCOOH-gluc; (E) MRM transition 317.2/193.1 of CBG; (F) MRM transition 331.6/193.3 of 11-OH-THC, Figure S2: Individual MRM transitions of DUS sample spiked at LOQ level. (A) MRM transition 318.4/196.2 of THC-D3; (B) MRM transition 315.4/193.3 of THC and CBD; (C) MRM transition 345.4/299.4 of THCCOOH; (D) MRM transition 521.5/345.0 of THCCOOH-gluc; (E) MRM transition 317.2/193.1 of CBG; (F) MRM transition 331.6/193.3 of 11-OH-THC, Figure S3: Individual MRM transitions of a real positive DUS sample. (A) MRM transition 318.4/196.2 of THC-D3; (B) MRM transition 315.4/193.3 of THC and CBD; (C) MRM transition 345.4/299.4 of THCCOOH; (D) MRM transition 521.5/345.0 of THCCOOH-gluc.

## Sample Availability

Samples of the compounds are available from the authors.

## References

[1] Velghe S., Troyer R.D., Stove C. (2018). Dried Blood Spots in Therapeutic Drug Monitoring and Toxicology. Expert Opin. Drug Metab. Toxicol..

[2] Moretti M., Freni F., Tomaciello I., Vignali C., Groppi A., Visonà S.D., Tajana L., Osculati A.M.M., Morini L. (2019). Determination of Benzodiazepines in Blood and in Dried Blood Spots Collected from Post-Mortem Samples and Evaluation of the Stability over a Three-Month Period. Drug Test. Anal..

[3] Moretti M., Freni F., Valentini B., Vignali C., Groppi A., Visonà S.D., Osculati A.M.M., Morini L. (2019). Determination of Antidepressants and Antipsychotics in Dried Blood Spots (DBSs) Collected from Post-Mortem Samples and Evaluation of the Stability over a Three-Month Period. Molecules.

[4] Moretti M., Visonà S.D., Freni F., Tomaciello I., Vignali C., Groppi A., Tajana L., Osculati A.M.M., Morini L. (2018). A Liquid Chromatography-Tandem Mass Spectrometry Method for the Determination of Cocaine and Metabolites in Blood and in Dried Blood Spots Collected from Postmortem Samples and Evaluation of the Stability over a 3-Month Period. Drug Test. Anal..

[5] Moretti M., Manfredi A., Freni F., Previderé C., Osculati A.M.M., Grignani P., Tronconi L., Carelli C., Vignali C., Morini L. (2021). A Comparison between Two Different Dried Blood Substrates in Determination of Psychoactive Substances in Postmortem Samples. Forensic Toxicol..

[6] Capiau S., Veenhof H., Koster R.A., Bergqvist Y., Boettcher M., Halmingh O., Keevil B.G., Koch B.C.P., Linden R., Pistos C. (2019). Official International Association for Therapeutic Drug Monitoring and Clinical Toxicology Guideline: Development and Validation of Dried Blood Spot-Based Methods for Therapeutic Drug Monitoring. Ther. Drug Monit..

[7] Berry H.K. (1959). Procedures for Testing Urine Specimens Dried on Filter Paper. Clin. Chem..

[8] Auray-Blais C., Lavoie P., Zhang H., Gagnon R., Clarke J.T.R., Maranda B., Young S.P., An Y., Millington D.S. (2012). An Improved Method for Glycosaminoglycan Analysis by Lc-Ms/Ms of Urine Samples Collected on Filter Paper. Clin. Chim. Acta.

[9] Breier A.C., Cé J., Coelho J.C. (2014). Correlation of the levels of glycosaminoglycans between urine and dried urine in filter paper samples and their stability over time under different storage temperatures. Clin. Chim. Acta.

[10] Antunes M.V., Niederauer C.G., Linden R. (2013). Development, validation and clinical evaluation of a dried urine spot method for determination of hippuric acid and creatinine. Clin. Biochem..

[11] Naritaka N., Suzuki M., Takei H., Chen H.-L., Oh S., Kaewplang P., Zhang C., Murai T., Kurosawa T., Kimura A. (2019). Use of Dried Urine Spots for Screening of Inborn Errors of Bile Acid Synthesis. Pediatr. Int..

[12] Schmidt J., Lindemann V., Olsen M., Cramer B., Humpf H.U. (2021). Dried Urine Spots as Sampling Technique for Multi-Mycotoxin Analysis in Human Urine. Mycotoxin Res..

[13] Michely J.A., Meyer M.R., Maurer H.H. (2017). Dried Urine Spots—A Novel Sampling Technique for Comprehensive Lc-Ms(N) Drug Screening. Anal. Chim. Acta.

[14] Lee Y., Lai K.K.Y., Sadrzadeh S.M.H. (2013). Simultaneous Detection of 19 Drugs of Abuse on Dried Urine Spot by Liquid Chromatography-Tandem Mass Spectrometry. Clin. Biochem..

[15] Pablo A., Breaud A.R., Clarke W. (2020). Automated Analysis of Dried Urine Spot (Dus) Samples for Toxicology Screening. Clin. Biochem..

[16] Scheidweiler K.B., Desrosiers N.A., Huestis M.A. (2012). Simultaneous Quantification of Free and Glucuronidated Cannabinoids in Human Urine by Liquid Chromatography Tandem Mass Spectrometry. Clin. Chim. Acta.

[17] Dong X., Li L., Ye Y., Zheng L., Jiang Y. (2016). Simultaneous Determination of Major Phytocannabinoids, Their Main Metabolites, and Common Synthetic Cannabinoids in Urine Samples by Lc-Ms/Ms. J. Chromatogr. B.

[18] White R.M. (2018). Instability and Poor Recovery of Cannabinoids in Urine, Oral Fluid, and Hair. Forensic Sci. Rev..

[19] Desrosiers N.A., Lee D., Scheidweiler K.B., Concheiro-Guisan M., Gorelick D.A., Huestis M.A. (2014). In Vitro Stability of Free and Glucuronidated Cannabinoids in Urine Following Controlled Smoked Cannabis. Anal. Bioanal. Chem..

[20] Stout P.R., Horn C.K., Lesser D.R. (2000). Loss of Thccooh from Urine Specimens Stored in Polypropylene and Polyethylene Containers at Different Temperatures. J. Anal. Toxicol..

