# Supplementary material for: Analysis of Cannabinoids and Metabolites in Dried Urine Spots (DUS)
Source: Molecules. 2021 Sep 2;26(17):5334. doi: 10.3390/molecules26175334 (PMC8434267; doi:10.3390/molecules26175334)

Supplementary Material Figure S1. Individual MRM transitions of blank DUS sample. (A) MRM transition 318.4/196.2 of THC-D3; (B) MRM transition 315.4/193.3 of THC and CBD; (C) MRM transition 345.4/299.4 of THCCOOH; (D) MRM transition 521.5/345.0 of THCCOOH-gluc; (E) MRM transition 317.2/193.1 of CBG; (F) MRM transition 331.6/193.3 of 11-OH-THC.

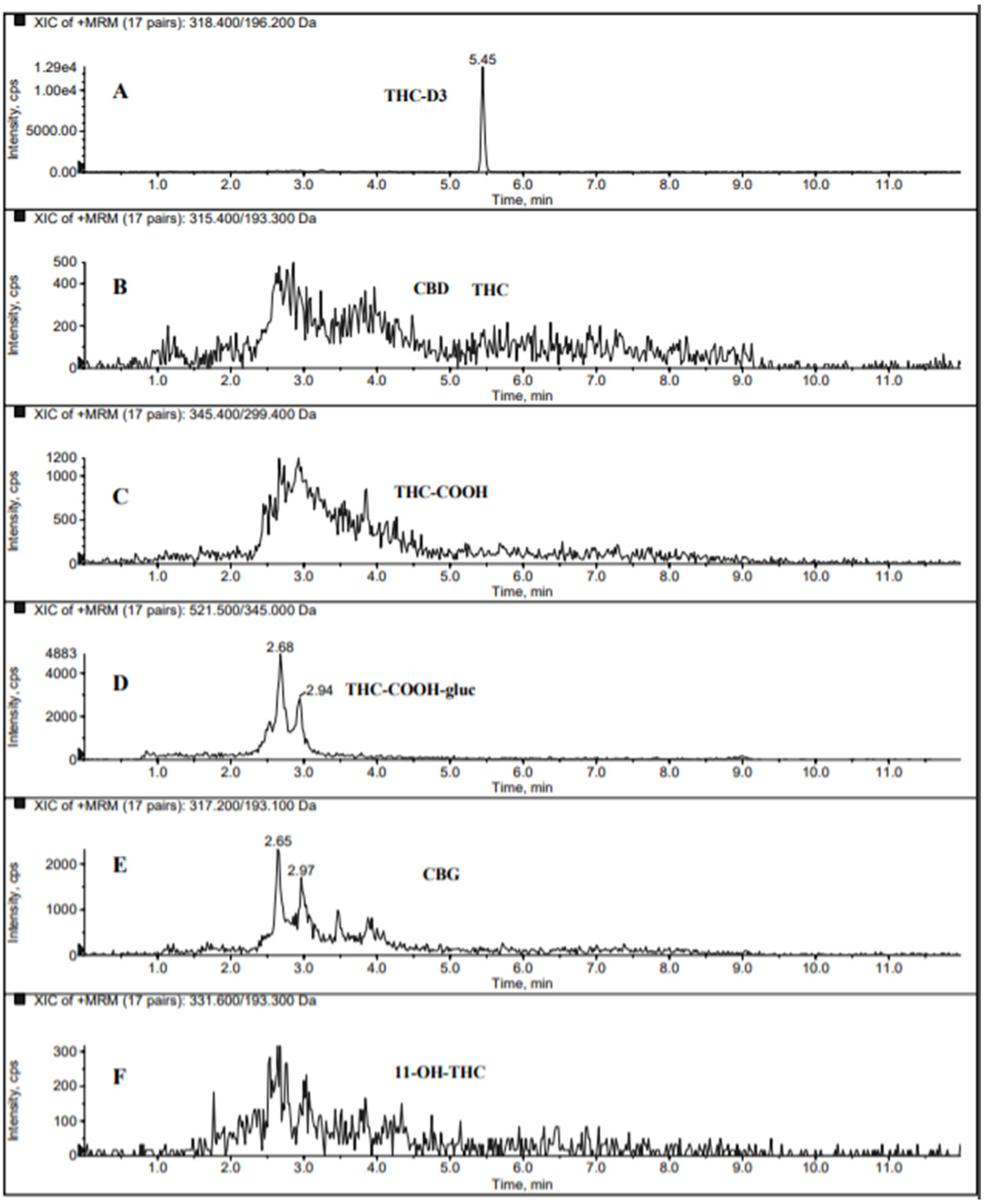

Supplementary Material Figure S2. Individual MRM transitions of DUS sample spiked at LOQ level. (A) MRM transition 318.4/196.2 of THC-D3; (B) MRM transition 315.4/193.3 of THC and CBD; (C) MRM transition 345.4/299.4 of THCCOOH; (D) MRM transition 521.5/345.0 of THCCOOH-gluc; (E) MRM transition 317.2/193.1 of CBG; (F) MRM transition 331.6/193.3 of 11-OH-THC.

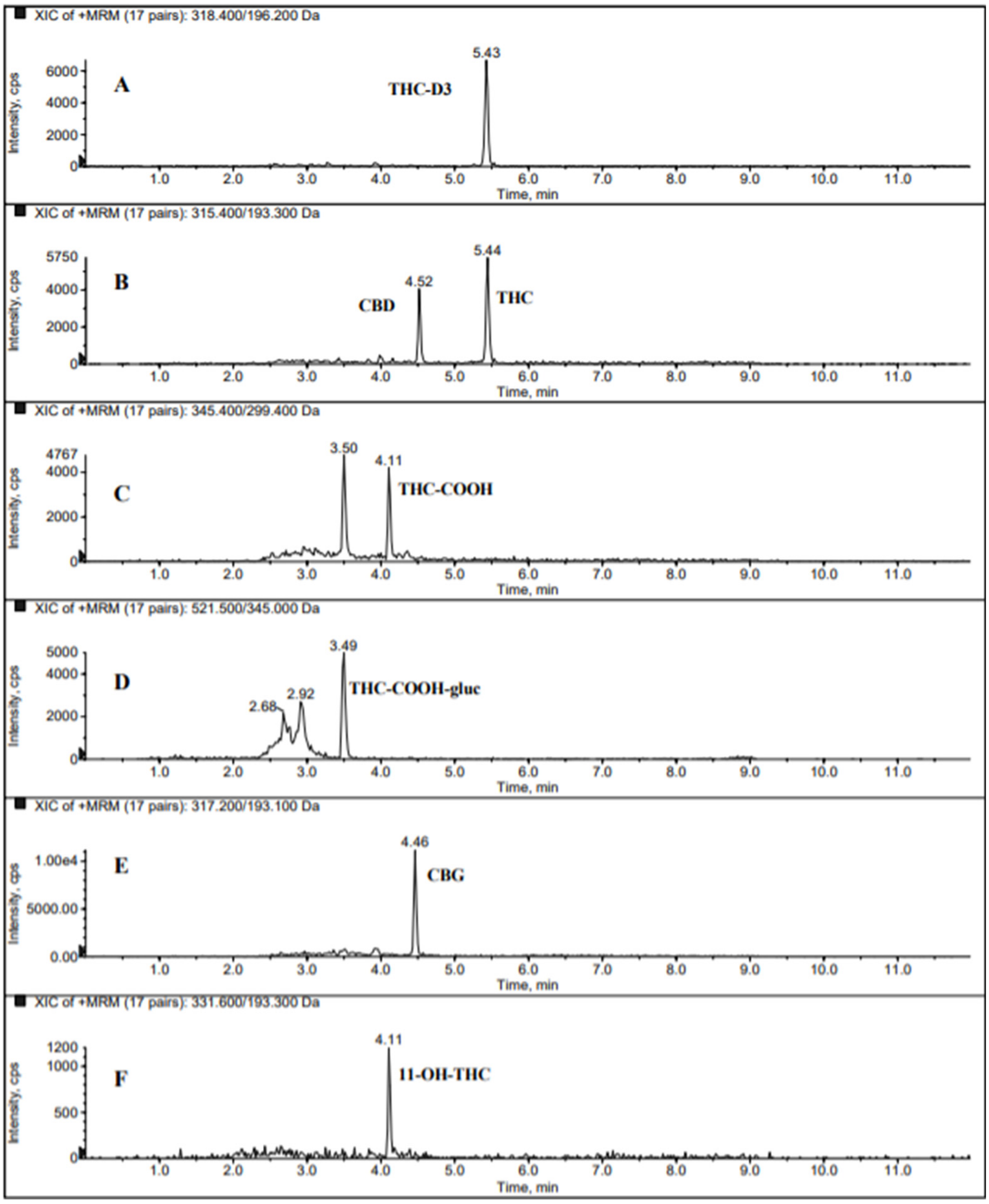

Supplementary Material Figure S3. Individual MRM transitions of a real positive DUS sample. (A) MRM transition 318.4/196.2 of THC-D3; (B) MRM transition 315.4/193.3 of THC and CBD; (C) MRM transition 345.4/299.4 of THCCOOH; (D) MRM transition 521.5/345.0 of THCCOOH-gluc.

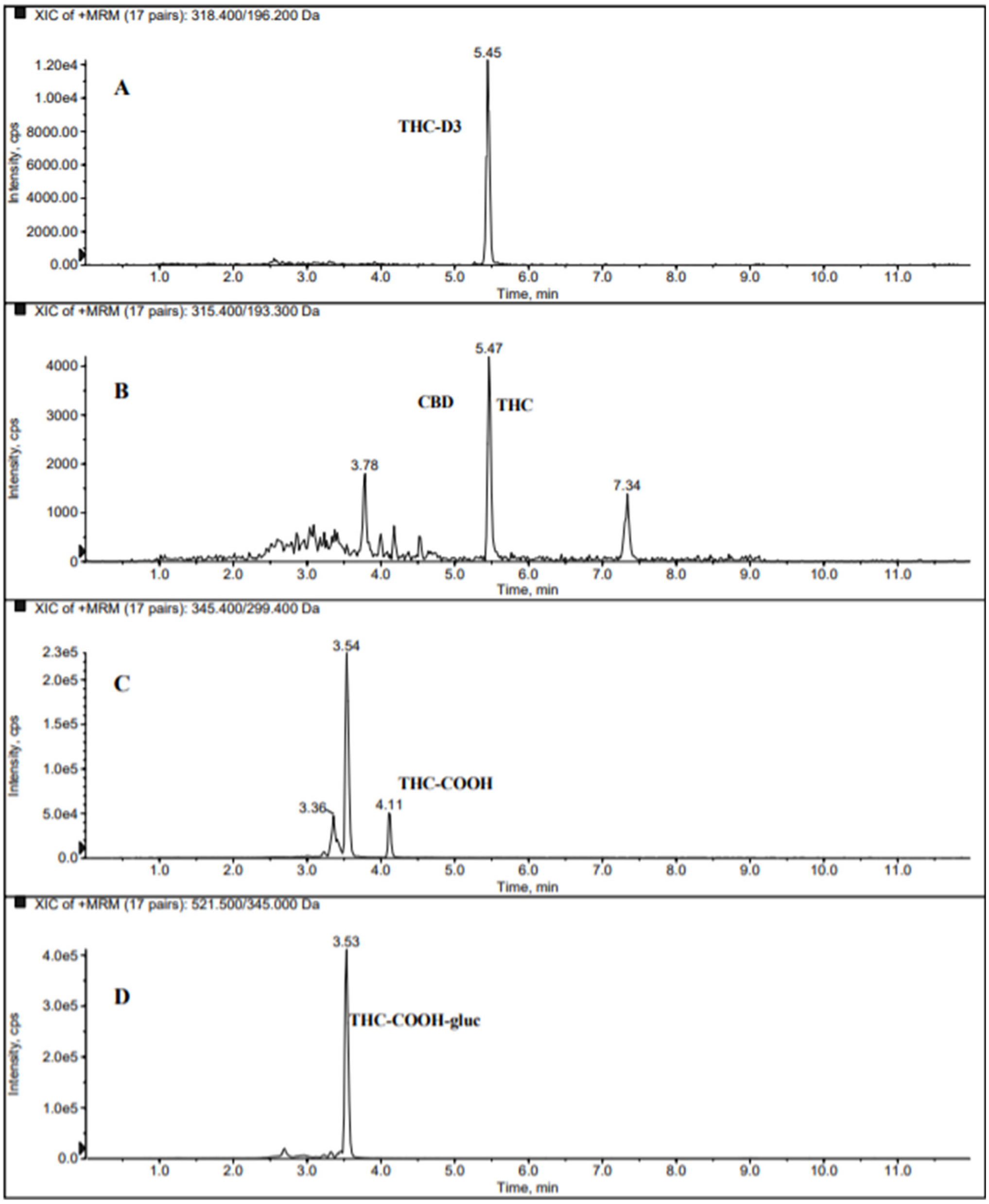

Supplement: Supplementary file 1 [file molecules-26-05334-s001.zip › molecules-1345035-supplementary.pdf]
